# Modeling extracellular stimulation of retinal ganglion cells: theoretical and practical aspects

**DOI:** 10.1088/1741-2552/acbf79

**Published:** 2023-03-13

**Authors:** Kathleen E Kish, Scott F Lempka, James D Weiland

**Affiliations:** 1 Department of Biomedical Engineering, University of Michigan, Ann Arbor, MI, United States of America; 2 Department of Anesthesiology, University of Michigan, Ann Arbor, MI, United States of America; 3 Department of Ophthalmology and Visual Science, University of Michigan, Ann Arbor, MI, United States of America; 4 BioInterfaces Institute, University of Michigan, Ann Arbor, MI, United States of America

**Keywords:** retinal stimulation, retinal ganglion cell, multi-compartment cable model, activating function

## Abstract

*Objective.* Retinal prostheses use electric current to activate inner retinal neurons, providing artificial vision for blind people. Epiretinal stimulation primarily targets retinal ganglion cells (RGCs), which can be modeled with cable equations. Computational models provide a tool to investigate the mechanisms of retinal activation, and improve stimulation paradigms. However, documentation of RGC model structure and parameters is limited, and model implementation can influence model predictions. *Approach.* We created a functional guide for building a mammalian RGC multi-compartment cable model and applying extracellular stimuli. Next, we investigated how the neuron’s three-dimensional shape will influence model predictions. Finally, we tested several strategies to maximize computational efficiency. *Main results.* We conducted sensitivity analyses to examine how dendrite representation, axon trajectory, and axon diameter influence membrane dynamics and corresponding activation thresholds. We optimized the spatial and temporal discretization of our multi-compartment cable model. We also implemented several simplified threshold prediction theories based on activating function, but these did not match the prediction accuracy achieved by the cable equations. *Significance.* Through this work, we provide practical guidance for modeling the extracellular stimulation of RGCs to produce reliable and meaningful predictions. Robust computational models lay the groundwork for improving the performance of retinal prostheses.

## 1. Introduction

Retinal prostheses aim to provide artificial vision for blind people, using an implanted neurostimulation device [1]. They have been used to treat profound vision loss caused by retinal degenerative diseases, such as retinitis pigmentosa and age-related macular degeneration [1]. The devices are implanted in or near the eye to target inner retinal neurons (ganglion and bipolar cells) that survive, even during late-stage disease [1]. Retinal prostheses have been shown to improve users orientation and mobility, and allow them to locate high-contrast objects [1]. However, the visual acuity possible with artificial vision remains limited, with a best-reported value of 20/400 [2]. To improve outcomes, it is critical to understand the fundamental mechanisms underlying the electrical excitation of retinal neurons. Computational models provide a tool to investigate how various anatomical and biophysical factors contribute to retinal activation, and to design improved stimulation paradigms and devices. In this work, we used a two-part technique to model the electrical stimulation of retinal tissue. We used finite element analysis to model the current flow from the stimulating electrodes. We then coupled the calculated electric fields with multi-compartment cable models, to capture the membrane dynamics of target neurons.

The most widely used family of models describing retinal ganglion cell (RGC) biophysics was initially developed by Fohlmeister, Miller and colleagues at the University of Minnesota [3–6]. This seminal model was based on whole cell patch clamp recordings from neurons in the tiger salamander retina [3]. They derived cable equations describing ion channel gating kinetics by fitting a model to experimental data, using the same mathematical structure as Hodgkin and Huxley [7]. In 2010, Fohlmeister *et al* adapted the original model to describe mammalian RGC membrane dynamics using patch clamp data from rat and cat retinal neurons [6]. Importantly, they included four ion channels in the RGC membrane (sodium, calcium, calcium-activated potassium, and delayed rectifier potassium). They used temperature as an independent variable to determine the ion channel distribution across various cellular regions and calculate experimental Q10 values.

The Fohlmeister cable equations have been the basis for hundreds of modeling projects. Despite their widespread use, documentation of RGC model structure and parameters is sparse and inconsistent. Model implementation can influence performance and simplifying assumptions may limit the significance of the model predictions. In this paper, we provide detailed instructions for creating a mammalian RGC cable model, and share all code necessary to replicate the model. Next, we investigated several practical aspects of modeling the extracellular stimulation of RGCs. A critical question is the extent to which the neuron’s three-dimensional (3D) shape will influence model predictions. We conducted sensitivity analyses to study how dendrite representation, axon trajectory, and axon diameter influence membrane dynamics and corresponding activation thresholds. Another practical consideration is how to maximize computational efficiency. When modeling a large number of neurons (e.g. a densely populated retina [8]), runtime remains a limiting factor in spite of modern computing resources. First, we optimized the spatial and temporal discretization of our cable model. Second, we applied several simplified threshold prediction theories to compare their prediction accuracy with our RGC cable model. The overall aim of this work was to provide shareable tools for modeling extracellular stimulation of RGCs, to investigate how morphometric factors influence model predictions, and to test several strategies for decreasing computation time.

## 2. Methods

### 2.1. RGC membrane dynamics

We defined the biophysical properties of our RGC cable model following previous work [6]. We set the temperature of the simulation environment to 37.1 °C. The cytoplasmic (axial) resistivity was 136.6 Ω cm and the membrane capacitance was 1 *µ*F cm^−2^ across all compartments [6]. The overall differential equation governing membrane voltage was:

\begin{align*}&amp;{C_{\text{m}}}\left( {\frac{{{\text{d}}V}}{{{\text{d}}t}}} \right) + {\bar g_{{\text{Na}}}}{m^3}h\left( {V - {E_{{\text{Na}}}}} \right) + \left( {{{\bar g}_{\text{K}}}{n^4} + {g_{{\text{K,Ca}}}}} \right)\left( {V - {E_{\text{K}}}} \right)\\ &amp; \quad + {\bar g_{{\text{Ca}}}}{c^3}\left( {V - {E_{{\text{Ca}}}}} \right) + {\bar g_{{\text{pas}}}}\left( {V - {E_{{\text{pas}}}}} \right) = {I_{{\text{stim}}}}\end{align*}

For the voltage-gated ion channels (Na^+^, K^+^, Ca^2+^), the channel state variables (*m, h, n, c*) are governed by equations of the form:

\begin{align*}\frac{{{\text{d}}x}}{{{\text{d}}t}} = - \left( {{\alpha _x} + {\beta _x}} \right)x + {\alpha _x}.\end{align*}

The voltage-dependent rate equations for each state variable are shown in table 1. From Fohlmeister *et al* (2010), the equations were adjusted by the Q10 values corresponding to 37.1 °C (see [6], table 2).

**Table 1.** Rate constants for voltage-gated ion channels [6].

Sodium (Na^+^)	
${\alpha _m} = \dfrac{{ - 3.136\left( {V + 35} \right)}}{{{{\text{e}}^{ - 0.1\left( {V + 35} \right)}} - 1}}$	${\beta _m} = 104.545{{\text{e}}^{\frac{{ - \left( {V + 60} \right)}}{{20}}}}$
${\alpha _h} = 2.091{{\text{e}}^{\frac{{ - \left( {V + 52} \right)}}{{20}}}}$	${\beta _h} = \dfrac{{31.365}}{{1 + {{\text{e}}^{ - 0.1\left( {V + 22} \right)}}}}$
Delayed rectifier potassium (K^**+**^)	
${\alpha _n} = \dfrac{{ - 0.110\left( {V + 37} \right)}}{{{{\text{e}}^{ - 0.1\left( {V + 37} \right)}} - 1}}$	${\beta _n} = 2.191{{\text{e}}^{\frac{{ - \left( {V + 47} \right)}}{{80}}}}$
Calcium (Ca^2+^)	
${\alpha _c} = \dfrac{{ - 1.568\left( {V + 13} \right)}}{{{{\text{e}}^{ - 0.1\left( {V + 13} \right)}} - 1}}$	${\beta _c} = 52.267{{\text{e}}^{\frac{{ - \left( {V + 38} \right)}}{{18}}}}$

**Table 2.** Nernst potentials (mV).

*E*_Na_	61.02
*E*_K_	−102.3
*E*_Ca_	$\frac{{RT}}{{2F}}\ln \left\{ {\frac{{{{\left[ {{\text{C}}{{\text{a}}^{2 + }}} \right]}_e}}}{{{{\left[ {{\text{C}}{{\text{a}}^{2 + }}} \right]}_i}\left( t \right)}}} \right\}$
*E*_pas_	−65.02

Unlike the other ion channels, the calcium-activated potassium channel (K_Ca_^+^) is ligand-gated according to the following equation:

\begin{align*}{g_{{\text{K,Ca}}}} = {\bar g_{{\text{K,Ca}}}}\frac{{{{\left( {{{\left[ {{\text{C}}{{\text{a}}^{2 + }}} \right]}_i}/{{10}^{ - 6}}{\text{M}}} \right)}^2}}}{{1 + {{\left( {{{\left[ {{\text{C}}{{\text{a}}^{2 + }}} \right]}_i}/{{10}^{ - 6}}{\text{M}}} \right)}^2}}}.\end{align*}

Calcium ion concentration is driven by a pump mechanism, with the equation, where *F* is Faraday’s constant (96 489 °C) and *r* is the depth (0.1 *µ*m) at which the calcium ion concentration [Ca^2+^]*_i_* is measured:

\begin{align*}\frac{{{\text{d}}{{\left[ {{\text{C}}{{\text{a}}^{{\text{2 + }}}}} \right]}_i}}}{{{\text{d}}t}} = \frac{{ - 3{I_{{\text{Ca}}}}}}{{2Fr}} - \frac{{\left( {{{\left[ {{\text{C}}{{\text{a}}^{2 + }}} \right]}_i} - {{10}^{ - 7}}{\text{M}}} \right)}}{{1.5{\text{ms}}}}.\end{align*}

Each ion has a unique Nernst potential, defined in table 2. These potentials are constant, except for calcium.

The maximum ion channel conductance values, listed in table 3, represent the ion channel density in each region. The RGC axon is subdivided into four distinct regions [6, 9]. The sodium channel band (SOCB) has an ion channel density 3–5× higher than the neighboring regions, making it prone to excitation [9, 10]. Since Fohlmeister *et al* defined a range of appropriate conductance values, we adopted exact parameters following Raghuram *et al* [11]. Other physical characteristics of each region (e.g. length, diameter) are provided in table 4.

**Table 3.** Regional maximum ion channel conductances (mS cm^−2^).

	${\bar g_{{\text{Na}}}}$	${\bar g_{\text{K}}}$	${\overline g _{{\text{Ca}}}}$	${\bar g_{{\text{K,Ca}}}}$	${\bar g_{{\text{pas}}}}$
Dendrites	60	35	1	0.17	0.1
Soma	60	35	0.75	0.17	0.1
Axon hillock	150	90	0.75	0.17	0.1
SOCB	420	250	0.75	0.11	0.1
Narrow region	150	90	0.75	0.2	0.1
Axon	100	50	0.75	0.2	0.1

**Table 4.** Length and diameter of retinal ganglion cell regions.

	Length (*µ*m)	Diameter (*µ*m)
Dendrites	*From 3D cell tracing* [12]	
Soma	20	20
Axon hillock	40	3
SOCB	40	3-0.8 taper
Narrow region	75	0.8
Axon	3000	1

We implemented our multi-compartment cable model and governing equations using NEURON v 7.7 in a Python simulation environment [13]. All code used to build the RGC cable model is online and freely available at: https://github.com/Kathleen-Kish/Retinal_Ganglion_Cell.

#### 2.1.1. Applying extracellular voltage

**Figure 1.**
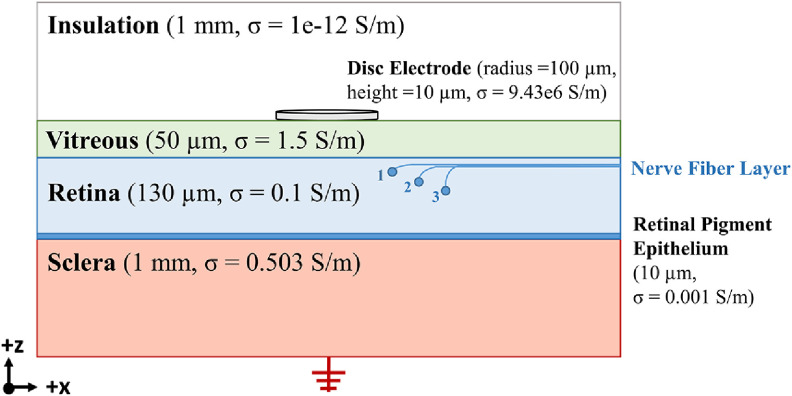
Finite element model configuration. The rectangular model had an overall size of 25 × 17 × 2.18 mm (figure not to scale). The cross-section shown is in the *x*–*z* plane. The schematic also shows a sample location for an RGC cable model with a shallow (1), medium (2), and deep (3) soma. All RGC axons extend in the +*x* direction, constituting the nerve fiber layer.


To predict the RGC response to extracellular stimulation, we used the finite element method (FEM) to determine the extracellular potential produced by the stimulating electrode at each compartment of the cable model (V_e_) [14]. The geometry of our simplified 3D model is shown in figure 1. We conducted finite element analysis in COMSOL Multiphysics Version 5.6 (Stockholm, Sweden) using the AC/DC electric currents (ec) module. This physics interface is used to compute the electric field, current, and potential in conductive media. For these simulations, we represented the active electrode as a 1 A current terminal (surface boundary condition), and assigned bulk tissue conductivities to each domain. We designated the bottom boundary of the sclera as an electrical ground (0 V). We obtained the tissue conductivities and layer thicknesses from prior work [15–17]. Using COMSOL, we generated a physics-controlled finite element mesh with extra fine element size. We used a quasi-static solver to calculate the electric potential (φ) distribution throughout the mesh. This solver employed the conjugate gradient method to solve Laplace’s equation, shown below:

\begin{align*}\nabla\cdot\,(\sigma\nabla\phi)=0\end{align*}

After solving the potential fields generated by the stimulation, we interpolated the spatially dependent FEM solution to find the extracellular potential at the center of each neuron compartment. These *V*_e_ values were exported from COMSOL as a .txt file and imported to Python. To couple the cable model with the applied extracellular potentials, we used the ‘extracellular’ mechanism in NEURON (xraxial = 1 × 10^9^ S cm^−1^, xg = 1 × 10^10^ S cm^−2^, and xc = 0 *µ*F cm^−2^). Since biological tissue conductivities are predominantly linear at retinal stimulation frequencies (5–100 Hz [1]), we scaled the extracellular potentials calculated for a 1 A current by the time-dependent stimulus pulse parameters, and integrated over time to calculate the membrane voltage response [18]. We chose electrode size and stimulus pulse parameters (biphasic, cathodic-first, 0.45 ms/phase) to match the Argus II device [19]. A version of our COMSOL model including the 3D geometry is posted on GitHub, and a full version including the mesh and solution is available upon request.

### 2.2. Cell morphometry

Naturally, RGCs exhibit substantial variations in both somatodendritic and axonal morphometry [20]. To study the effects of cell morphometry modifications on model behavior, we conducted several sensitivity analyses.

#### 2.2.1. Dendritic arbor

First, we studied the importance of including a dendritic arbor as part of an RGC cable model when modeling extracellular stimulation. An RGC model published by Schiefer and Grill does not include any dendrites [21]. On the other hand, many recent publications include a full branched dendritic morphology traced from *ex vivo* images [6, 22–25]. Werginz suggests that using a simplified equivalent cylinder to represent the dendrites produces nearly identical results as a more complex model [26].

**Figure 2.**
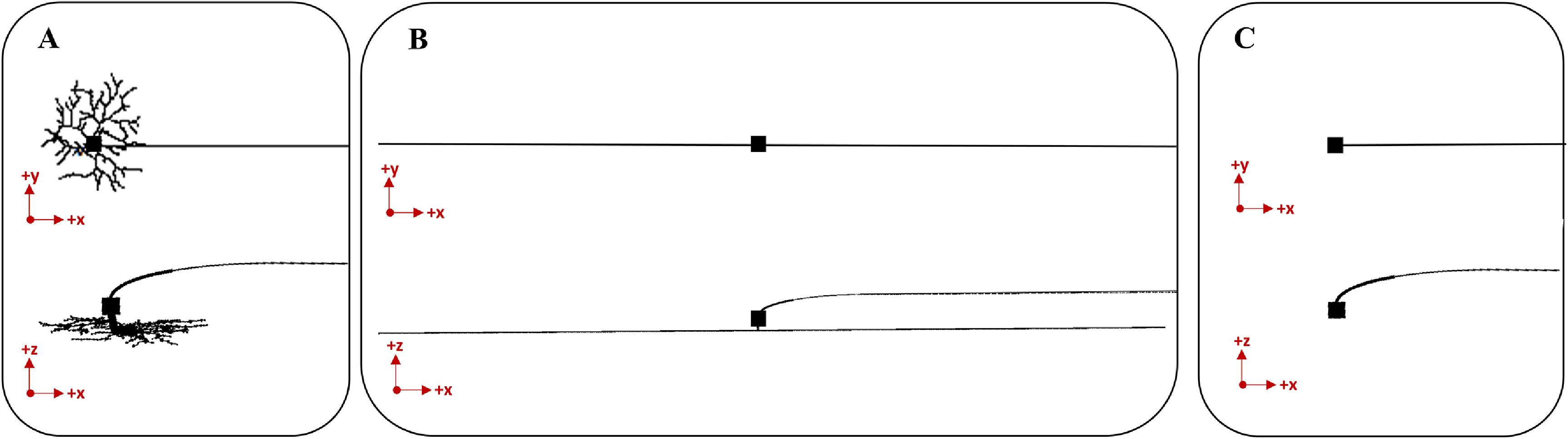
Multiple representations of the dendritic arbor in an RGC cable model. (a) Full branched morphology traced from a human cell [12]. The dendritic field size is 168 × 183 *µ*m in the *x*–*y* plane. (b) Equivalent cylinder model. The cylinder extends for 810 *µ*m in both the +*x* and −*x* direction after descending 10 *µ*m in the −*z* direction. (c) Simple model with no dendrites.

To reconcile the conflicting approaches in previous studies, we designed an experiment comparing an RGC model with a full branched dendritic arbor, an RGC model with an equivalent cylinder used to represent the dendrites, and an RGC model with no dendrites (figure 2). To identify a suitable dendrite tracing, we searched the Neuromorpho database with keywords ‘human’ and ‘ganglion’ [27]. Two publications were identified, one containing five intrinsically photosensitive RGCs [28] and the other containing forty-seven RGCs from the mid and peripheral retina [12]. We selected a single cell tracing (ID: NMO_110421) with a mid-size dendritic field (168 × 183 *µ*m). This is consistent in size with a parasol cell found 2–4 mm from the fovea [29] and would be reasonably targeted by electrodes implanted in the mid-peripheral region [30]. To create the equivalent cylinder model, we used a short vertical dendritic section (length = 10 *µ*m, diameter = 4 *µ*m) attached to a longer horizontal dendritic section (length = 1620 *µ*m, diameter = 2 *µ*m), following the methods described in prior work [26]. The equivalent cylinder approximates the somatodendritic membrane as a lumped impedance by matching the surface area of the original model (11 560 *µ*m^2^) [31].

We compared the response of the models to extracellular stimulation from a disc electrode [32]. We placed the electrode at a height of 50 *μ*m above the retina, which is within the range of electrode-retina distances for current clinical devices [30, 33]. We moved the electrode in a two-dimensional grid (1 × 1 mm) above the soma, with a 50 *µ*m step-size. We calculated the action potential threshold at each electrode location using a bisection algorithm (with convergence of 0.1 *µ*A).

#### 2.2.2. Axon trajectory

Next, we investigated the RGC axon pathway as it ascends from the soma and enters the nerve fiber layer. Anatomical studies show that axon trajectories vary among cells [34]. Prior models have made assumptions about the axon; for example, that it follows a 90° circular arc [21, 35] or ascends linearly [9, 36].

**Figure 3.**
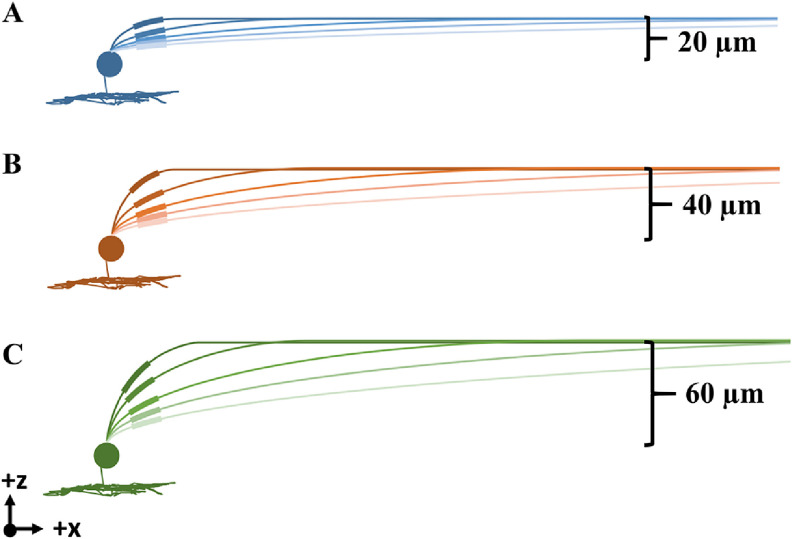
Multiple representations of the RGC axon as it ascends from the soma to the nerve fiber layer. The sodium channel band is plotted with a thicker line for visualization. (a) Shallow soma, 35 *µ*m below retinal surface and 20 *µ*m below nerve fiber layer. (b) Medium soma, 55 *µ*m below retinal surface. (c) Deep soma, 75 *µ*m below retinal surface.

For this analysis, we systematically explored the influence of RGC axon trajectory on activation threshold. We calculated the path of an ellipse with one vertex at the soma and the other at the nerve fiber layer [37]. We adjusted the distance between ellipse vertices to create variable curvature and steepness (figure 3). We tested RGCs with multiple soma depths (35, 55, 75 *µ*m) to represent natural variations [38]. We compared the response of the models to extracellular stimulation from a disc electrode. We placed the electrode at a height of 50 *μ*m above the retina, and moved it horizontally along the length of the axon. We calculated action potential threshold at each electrode location using a bisection algorithm (with convergence of 0.1 *µ*A).

#### 2.2.3. Axon diameter

As described in table 4, RGC axons are divided into several regions that vary in both physical dimensions and ion channel densities. Early studies identified that RGC axons have a narrow segment with an average length of 75 *µ*m, surrounded by a larger diameter region on either side [39]. More recently, Fried *et al* used immunochemical staining to identify an SOCB adjacent to the narrow segment, densely populated with voltage-gated Na^+^ channels, that is on average 40 *µ*m long [10]. Experimental thresholds are lowest when the stimulating electrode is placed over this band [10].

Axon diameter in all four regions has been inconsistent in prior computational models. When measured anatomically, RGC axon diameter has been generally shown to scale with cell size [6, 11]. We conducted a sensitivity analysis to investigate the effect of axon diameter on membrane dynamics and action potential threshold for extracellular RGC stimulation. We placed the disc electrode in three locations: directly above the soma, above the SOCB (150 *µ*m offset in the +*x* direction), and above the distal axon (500 *µ*m offset in the +*x* direction). We adjusted the axon hillock diameter between 2–4 *µ*m and the narrow region diameter 0.6–1 *µ*m [11]. We tapered the SOCB diameter to connect these two regions smoothly [9]. In all simulations, we recorded membrane voltage at all compartments to classify the action potential propagation behavior. We calculated action potential threshold at each electrode location using a bisection algorithm (with convergence of 0.1 *µ*A).

### 2.3. Model run time

In general, more complex models require higher computation time. To maximize efficiency, it is beneficial to find the simplest model that produces reliable predictions. We investigated two strategies for improving the run time of our RGC cable model. First, we conducted a sensitivity analysis to identify the minimum temporal and spatial resolutions necessary to provide consistent predictions. Second, we examined the ability of simplified techniques, such as the activating function (AF), to predict activation threshold.

#### 2.3.1. Spatial and temporal discretization

Multi-compartment cable models predict a spatiotemporally continuous solution for the membrane voltage by solving at a finite number of points in space (sections) and time. NEURON discretizes time and space to solve the relevant partial differential equations using a backward Euler integration method [40]. As a result, the integration time step (Δ*t*) and the spatial interval between sections (Δ*x*) both contribute to the solution accuracy. In general, the runtime of a cable model is directly proportional to the product of Δ*t* and Δ*x* [40].

To establish a ‘ground truth’ solution, we set Δ*x* = 1 *µ*m and Δ*t* = 1 *µ*s and calculated the action potential threshold using a bisection algorithm. From there, we incrementally increased the value for Δ*x* until activation threshold changed by more than 0.1 *µ*A, documenting the change in computation time. We adjusted Δ*x* independently for each cell region and tested multiple electrode positions to ensure robust results. Then, we similarly increased the value for Δ*t* incrementally, documenting the change in activation threshold and computation time.

#### 2.3.2. AF for threshold prediction

**Figure 4.**
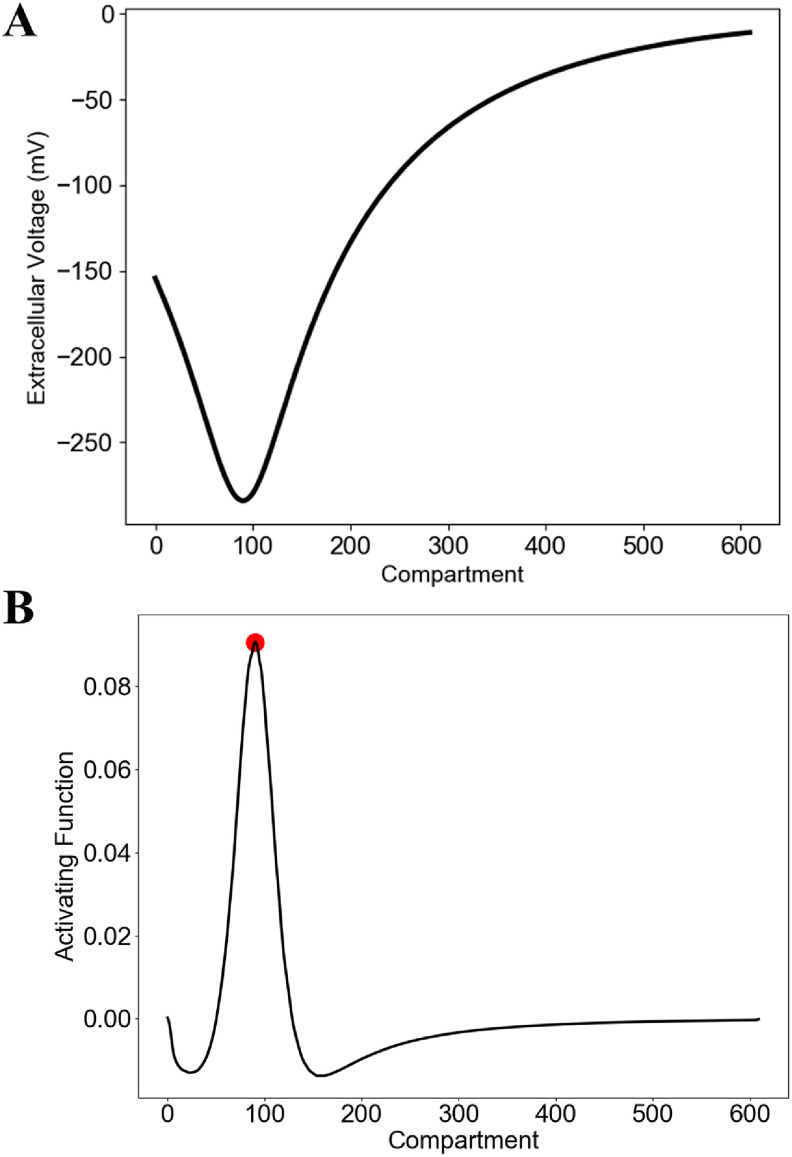
(a) Example of the extracellular potential (*V*_e_) generated along an RGC cable model during epiretinal stimulation with a disc electrode. Extracellular potentials are negative because the cathodic phase of the pulse causes activation. Compartment 0 represents the soma and compartment 610 represents the end of the axon; dendritic compartments are not shown. (b) Activating function (AF), calculated as the second spatial derivative of *V*_e_ along the RGC. The red dot represents the AF peak, hypothesized to be a predictor for the site of action potential initiation. The neuron’s location with respect to the electrode is shown in figure 5.

The second spatial derivative of extracellular potential along an unmyelinated axon, called the AF (AF = *∂^2^V*_e_ */∂x^2^*), drives a neuron’s response to an applied stimulus [41, 42]. The maximum value of the AF represents the point of peak depolarization on the cell membrane, thus, the most probable action potential initiation site (figure 4). The AF has also been used as a predictor for threshold [43–46]. This approach represents a significant reduction in computational demands because it does not require solution of the RGC membrane voltage.

While the AF approach is appropriate for a uniform axon, its validity remains unclear for neurons with non-uniform ion channel density and physical dimensions, such as RGCs. Werginz *et al* found that while the AF predicts the RGC membrane’s initial response to stimulation, uneven axial current flow during the stimulus pulse can limit its relevance [36]. Esler *et al* suggested using a weighted AF (AF_w_) to linearly approximate the cellular integration of transmembrane currents [46]. In this framework, the AF value at each neural compartment (ƒ_n_) is multiplied by a weight value (*w_n_*) representing its influence on the SOCB. The authors found that the sum (∑ƒ_n_·*w_n_*) across a certain number (*n*) of compartments could accurately predict RGC firing behavior [46].

We tested the ability of both the AF and weighted AF (AF_w_) to predict threshold for our RGC cable model. We used two methods to calculate the AF. First, we used Python’s gradient operator to calculate the second spatial derivative of the applied potential field along the trajectory of the RGC. To produce a smooth function with this technique, we discretized the finite element tetrahedral mesh with a cubic (third-order) shape function in COMSOL. Secondly, we implemented a passive RGC model in NEURON and used the ‘i_membrane’ variable as a proxy for the AF. This variable measures the net transmembrane current density for a given compartment. The transmembrane current (mA cm^−2^) in response to the applied extracellular potential field after one time step (*t* = 5 *µ*s) is proportional to the AF, while also incorporating differences in axial resistance (*R*_a_) caused by non-uniform section diameter. Finally, to calculate the weighted AF (AF_w_), we followed the methods described by Esler *et al* [46]. We calculated individual compartment weights (*w_n_*) by finding the relative SOCB depolarization resulting from injecting 1 nA current into each compartment, using a passive model. We tested the sum (∑ƒ_n_·*w_n_*), varying *n* = 1 to *n* = 50 compartments and ordering compartments from highest to lowest weight [46].

**Figure 5.**
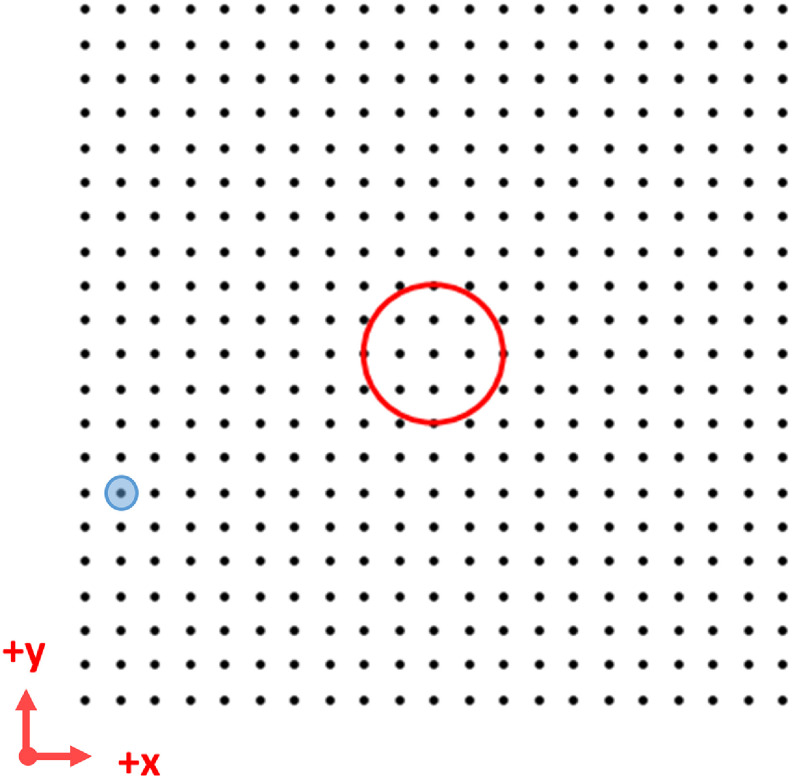
Position of RGC somata (black) beneath the stimulating electrode (red). Cell bodies are spaced 50 *µ*m apart to span a 1 × 1 mm retinal area. Axons extend in the +x direction, as above. The RGC used as an example in figure 4 is highlighted blue.

To determine the accuracy of these simplified threshold predictors, we calculated their value for 441 uniquely positioned RGCs (figure 5). We used full cable model solutions to determine the threshold electrode current amplitude and corresponding extracellular potential along the RGC. Then, we defined the AF threshold as the AF peak when applying the threshold *V*_e_ vector. For a reliable predictor, AF threshold should fit an exponential curve based on electrode-axon distance [43, 44]. We quantified prediction accuracy by calculating the coefficient of determination (*R* ^2^) for the exponential curve. We calculated electrode-RGC distance from the center of the electrode to the center of the soma.

## 3. Results

### 3.1. Dendritic arbor representation influences activation threshold map

**Figure 6.**
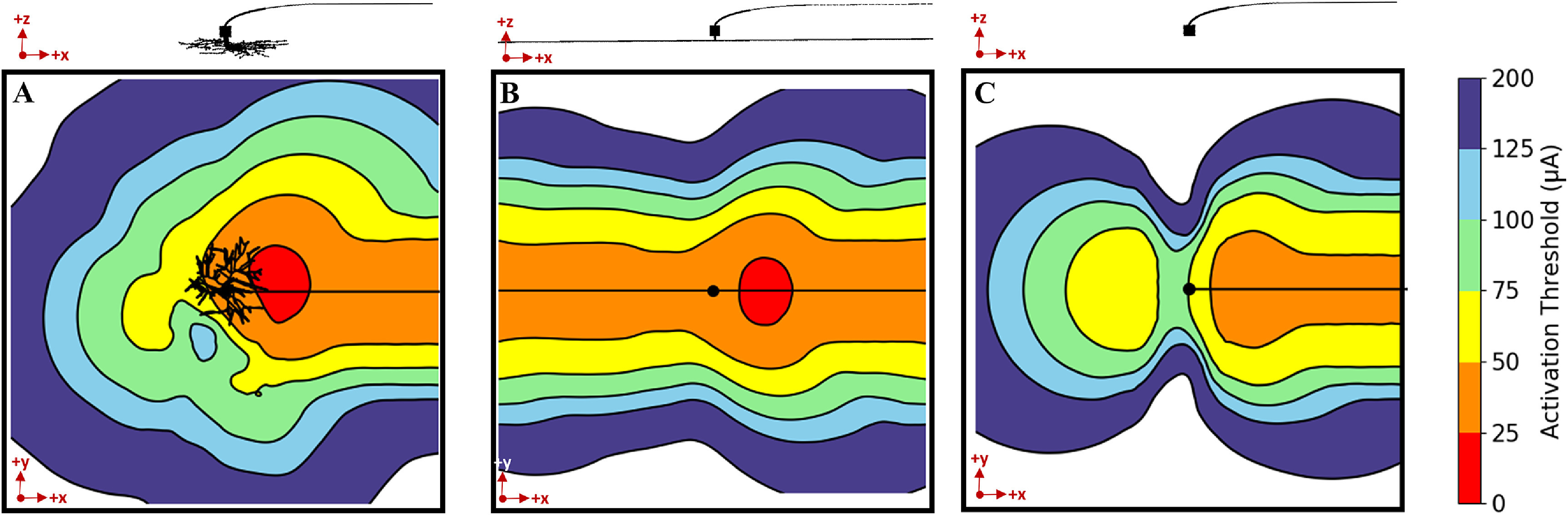
Threshold maps for various dendritic arbor representations: (a) Full-branched morphology traced from a human cell, (b) equivalent cylinder dendritic model, (c) simple model with no dendrites. Threshold values (*µ*A) were measured by moving the stimulating electrode in a 1 × 1 mm grid above the RGC, with 50 *µ*m step-size. Results are shown in the x–y plane, which is parallel to the disc electrode.

To characterize the effects of dendritic arbor on activation, we built three versions of an RGC cable model: (1) with a full-branched dendritic morphology traced directly from a human cell, (2) with an equivalent cylinder used to represent the dendrites, and (3) a simplified model with no dendrites (see figure 2). We compared the response of each cell model to extracellular stimulation. Figure 6 shows the distribution of activation thresholds (*µ*A) in response to a biphasic stimulus pulse, when a disc electrode was moved in a 1 × 1 mm grid above each cell model. Compared to the full branched morphology (figure 6(a)), the equivalent cylinder representation (figure 6(b)) predicted the same absolute minimum threshold (21.7 ± 0.1 *µ*A) located above the SOCB. However, the elongated dendritic geometry created a low threshold region spatially overlapping the equivalent cylinder. Removing the dendrites altogether (figure 6(c)) increased the threshold magnitude (absolute minimum: 26.4 ± 0.1 *µ*A), especially when the electrode was near the soma. This model lacks the active ion channels on the dendritic membrane, which can integrate the voltage produced by the electrode and increase the overall excitability of the neuron. Based on these results, we used the full-branched dendritic morphology for our remaining analyses.

### 3.2. Axon curvature modulates activation threshold profile

**Figure 7.**
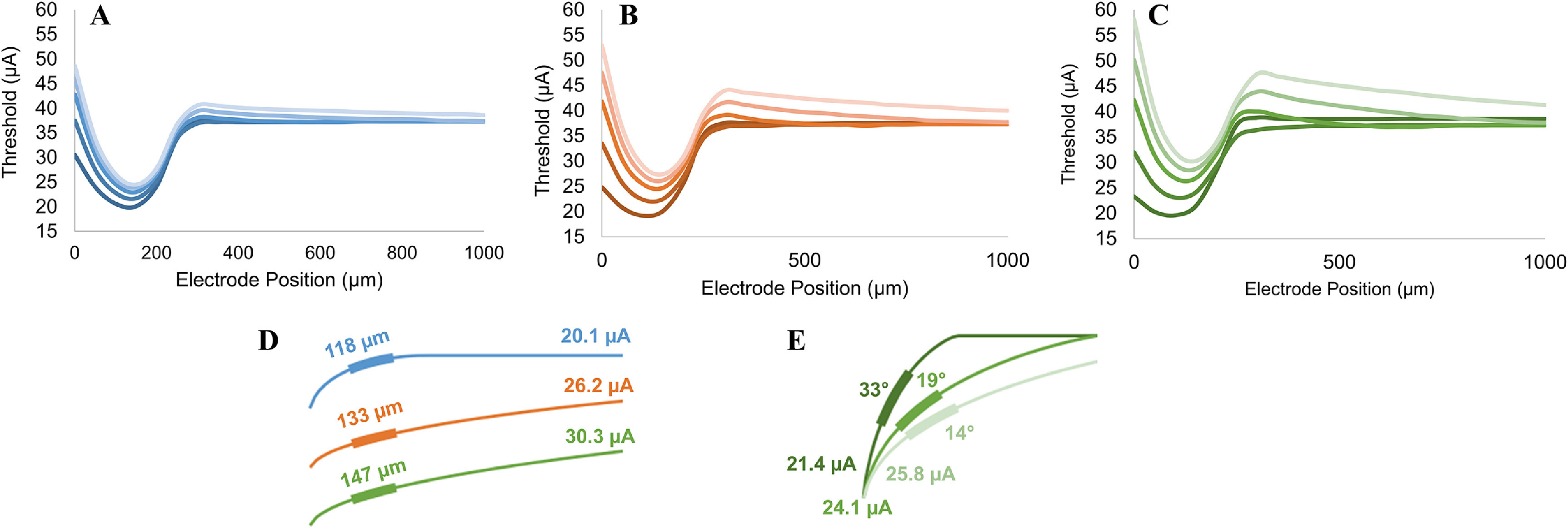
Threshold changes associated with varying axon trajectory for (a) shallow, (b) medium and (c) deep cell bodies. The plots show threshold (*µ*A) as the stimulating electrode shifts horizontally along the length of the RGC axon. The soma is located at 0 *µ*m, and the axon extends in the +x direction. Steeper trajectories are associated with darker shades; see figure 3 for more details. (d) For three RGC axons where the SOCB angle was 6°, the effect of SOCB-electrode distance on threshold. (e) For three axons where the SOCB-electrode distance was 140 *µ*m, the effect of SOCB angle on threshold. Note that SOCB angle refers to incline in the x–z plane, for a line drawn between the first and last compartment, where 0° is horizontal and we calculated the SOCB-electrode distance from the center of the disc electrode to the center of the SOCB. Figures (d) and (e) are not to scale.

We altered our RGC cable model by creating a series of elliptical axon trajectories with variable curvature and steepness, including several soma depths (see figure 3). Again, we compared their response to biphasic extracellular stimulation with a disc electrode. Figure 7 shows the profile of activation thresholds (*µ*A) as we shifted the stimulating electrode horizontally along the length of each axon. The minimum threshold always occurred when the electrode was above the SOCB. In general, steeper trajectories with sharper curvature had lower activation thresholds, particularly when the electrode was near the soma. The maximum threshold decrease (between gradual and steep trajectories) was 37%, 53%, and 60% for somata at depths of 35, 55, and 75 *µ*m, respectively.

Steeper trajectories may have lower activation thresholds for two reasons: smaller SOCB-electrode distance and greater SOCB angle. Both factors influence the electric field gradient across the SOCB, which drives activation. To separate these effects, we looked at two scenarios. First, we identified axons with an SOCB angle of 6°, and varying SOCB-electrode distance (figure 7(d)). Increasing the SOCB-electrode distance systematically increased threshold. Then, we identified axons with an SOCB-electrode distance of 140 *µ*m, and varying SOCB angle (figure 7(e)). Increasing SOCB angle systematically decreased threshold. This supports the claim that threshold decrease for steep trajectories is due to a combination of SOCB-electrode distance and SOCB angle.

### 3.3. Axon diameter affects action potential propagation

**Figure 8.**
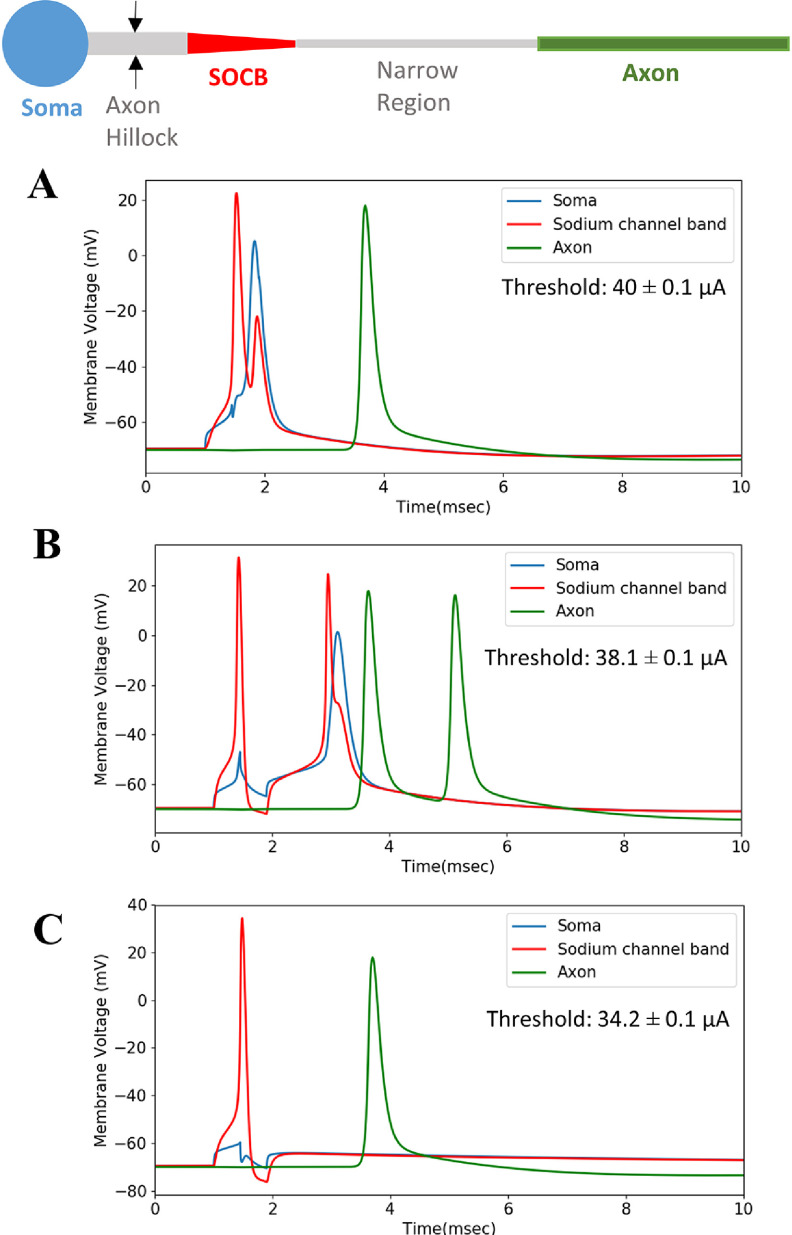
Axon hillock diameter (black arrows) affects action potential propagation. The biphasic stimulation pulse is applied at 1 ms. In all cases, the action potential initiates in the SOCB. (a) When the axon hillock has a 4 *µ*m diameter, the action potential propagates in both directions. (b) When the axon hillock has a 3 *µ*m diameter, we observe an ‘echo spike’ in the axon. (c) When the axon hillock has a diameter of 2 *µ*m, the action potential fails to invade the soma. For these simulations, the narrow region diameter was 0.8 *µ*m.

The final morphometry modification to our model was to change axon diameter (specifically in the axon hillock and narrow region) and evaluate action potential propagation. First, we adjusted the axon hillock diameter between 2 and 4 *µ*m. Figure 8 shows the membrane voltage dynamics of our model as we decreased axon hillock diameter. In this analysis, we applied the threshold stimulus pulse with a disc electrode directly above the soma. When the axon hillock diameter was 4 *µ*m, the action potential propagated initiated in the SOCB and propagated in both directions. When the axon diameter was 3 *µ*m, there was a prolonged latency before the action potential invaded the soma. This provided time for the SOCB membrane to repolarize and experience a secondary ‘echo spike’, which propagated down the axon. When the axon hillock diameter was 2 *µ*m, the action potential could not depolarize the somatodendritic membrane. The same behavior occurred when the stimulating electrode was located above the SOCB (150 *µ*m offset) or distal axon (500 *µ*m offset). As shown in figure 8, there was a minor decrease in activation threshold with axon hillock diameter when the electrode was above the soma. No change in threshold was observed when the electrode was above the SOCB (threshold: 22.5 ± 0.1 *µ*A) or distal axon (threshold: 38.1 ± 0.1 *µ*A).

Adjusting the narrow region diameter between 0.6 and 1.0 *µ*m caused analogous trends in the model. Action potentials propagated bi-directionally only when the narrow region diameter was greater than 0.8 *µ*m. Narrow region diameter had no influence on activation threshold. In some situations, increasing Na^+^ and K^+^ conductance in the narrow region could counteract a smaller diameter. For example, setting ${\bar g_{{\text{Na}}}}$ = 250 mS cm^−2^ and ${\bar g_{\text{K}}}$ = 125 mS cm^−2^ allowed for bidirectional propagation, even with a 0.6 *µ*m narrow region.

### 3.4. Optimized spatial and temporal discretization

At first, we built our multi-compartment cable model with a compartment length of 1 *µ*m and integrated with a time step of 1 *µ*s. We incrementally decreased the spatial and temporal resolution, measuring the change in activation threshold and computation time. Table 5 shows the spatial resolution of our final model by region. The ‘ground truth’ model contained 4260 sections, while the reduced model had 960 sections. Computation was 7.4 times faster for the spatially optimized model, with no differences in predicted threshold (±0.1 *µ*A).

**Table 5.** Spatial resolution of optimized model by region.

	Optimized section length (*µ*m)
Dendrites	10
Soma	4
Axon hillock	5
SOCB	5
Narrow region	5
Axon	5

Table 6 summarizes the effects of increasing the integration time step on simulation time required to calculate threshold for one neuron. We chose a value of Δ*t* = 5 *µ*s for our final model. Overall, performing this sensitivity analysis allowed us to solve the RGC cable equations 12.75 times faster, with a negligible influence on activation threshold.

**Table 6.** Influence of integration time step.

Timestep (*µ*s)	Threshold change (%)	Run time (min)
1	0	43.4
2	0	21.7
5	0	8.7
10	1.12	4.6
20	3.60	2.2
50	10.56	1.1

### 3.5. Simplified threshold predictors fall short of full cable model solutions

**Figure 9.**
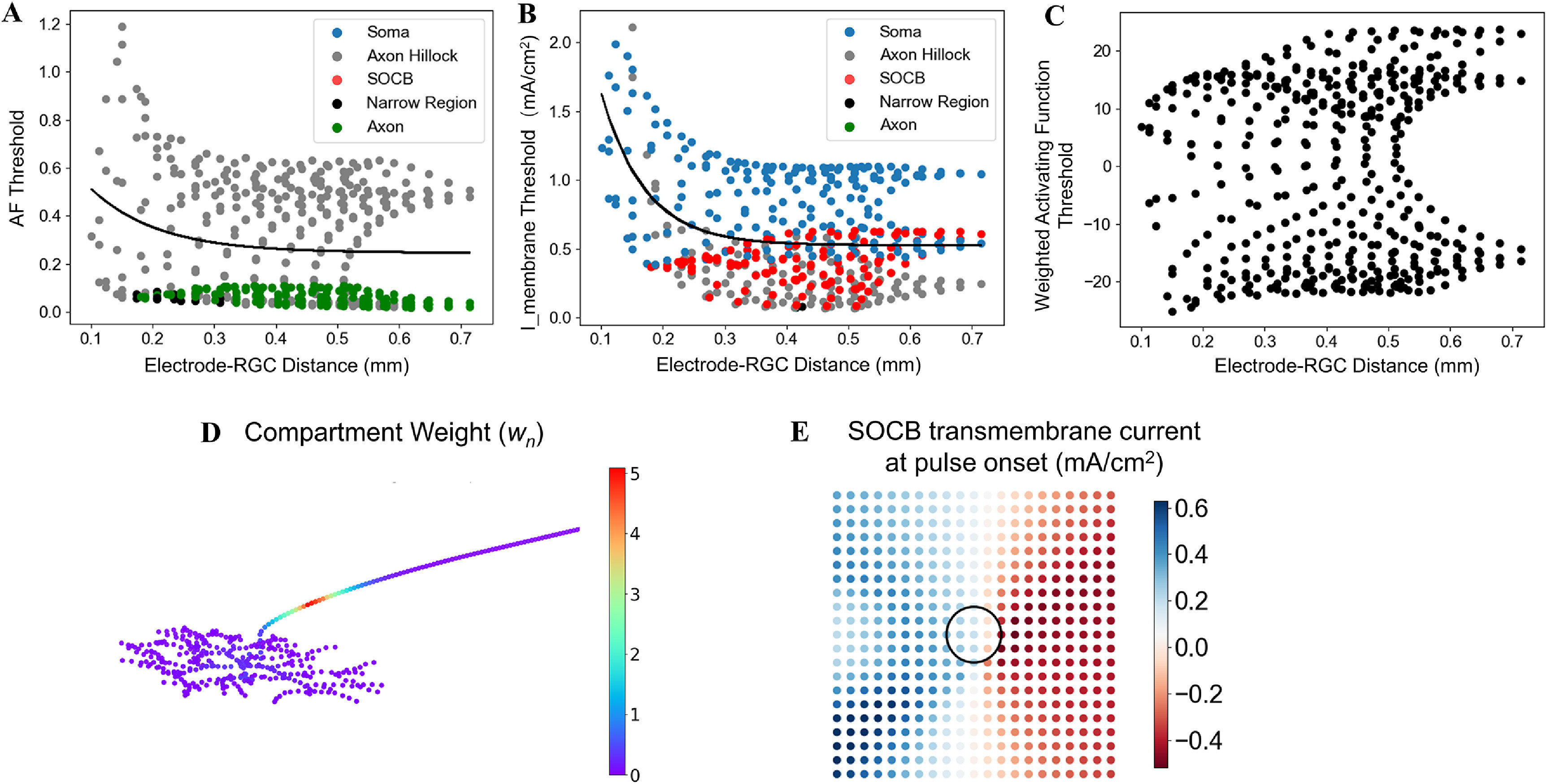
(a) AF threshold versus electrode-RGC distance, points are color-coded based on the spatial location of the AF peak. The black line shows the exponential fit: *y* = 0.67×10^−9.23x^ + 0.25, *R* ^2^ = 0.04. (b) Instantaneous transmembrane current threshold versus electrode-RGC distance, points are color-coded based on the spatial location of the peak. The black line shows the exponential fit: *y* = 4.71×10^−14.4x^ + 0.52, *R* ^2^ = 0.19. (c) Weighted activating function threshold (∑ƒ_n_·w_n_) versus electrode-RGC distance, *n* = 25. (d) Neuron compartment centers, color-coded by compartment weight (w_*n*_). (e) Instantaneous transmembrane current at the SOCB at pulse onset. Each dot represents a single RGC soma, as in figure 5. The magnitude and polarity (indicated by the color) varies with neuron location.

We assessed both an AF approach and a weighted AF (AF_w_) approach to reduce the computational demands required to predict threshold. We compared these simplified approaches to the threshold predictions generated with the full RGC cable model. First, we calculated the second spatial derivative by applying Python’s gradient operator twice to the extracellular potentials along the neuron. We calculated the AF threshold and spatial location of the AF peak for 441 uniquely positioned RGCs. Figure 9(a) shows AF threshold plotted against electrode-RGC distance. The exponential fit had an equation of *y* = 0.67 × 10^−9.23*x*^ + 0.25 with *R* ^2^ = 0.04. The low coefficient of determination indicates poor threshold prediction accuracy. Figure 9(a) also indicates the spatial location of the AF peak using color. The AF peak was most often located at the axon hillock, due to its changing orientation with respect to the soma (e.g. soma aligned with *z*-axis, axon hillock begins shifting in *x*-direction). However, the peak occurred in the axon when it passed directly beneath the disc electrode. The distribution of peak locations contradicts the cable model, in which the action potential always initiated in the SOCB.

Using transmembrane current (mA cm^−2^) after one time step (*t* = 5 *µ*s) as a substitute for the AF only produced modest improvements in prediction accuracy. We calculated the threshold and spatial location of the peak transmembrane current for 441 uniquely positioned RGCs. Figure 9(b) shows ‘i_membrane’ threshold plotted against electrode-RGC distance. The exponential fit had an equation of *y* = 4.71×10^−14.4*x*^ + 0.52 with *R* ^2^ = 0.19. By accounting for non-uniform section diameter, the instantaneous transmembrane current incorporates differences in axial resistance (*R*_a_) and provides a better estimate of the AF peak. The peak was more likely to occur in the large diameter soma and less likely to occur in the narrow axon. However, the AF peak was still only located in the SOCB 30% of the time with this method, which does not agree with the cable model.

The AF_w_ approach involved scaling the AF by specified compartment weights and finding the sum across *n* compartments (∑ƒ_n_·*w_n_*), ordering compartments from highest to lowest weight [40]. We first derived compartment weights (*w_n_*) by finding the SOCB depolarization resulting from injecting 1 nA current into each compartment, using a passive model, and normalizing [46]. Figure 9(d) shows the resulting weights, coded by color. We calculated AF_w_ across *n* = 1–50 compartments for 441 uniquely positioned RGCs at threshold. Figure 9(c) shows weighted AF threshold (∑ƒ_n_·*w_n_*) plotted against electrode-RGC distance when *n* = 25. Notably, this approach generated both positive and negative values that were largely dependent upon the AF polarity at the SOCB. The polarity varied based on the neuron’s location with respect to the stimulating electrode (figure 9(e)). As a result, we did not see a consistent value emerge at threshold, no matter how many compartments were included in the sum.

## 4. Discussion

This work had three main outcomes. We provided a functional guide for modeling extracellular RGC stimulation. We described how morphometric factors influence model predictions, adding to the prior work. Finally, we determined temporal and spatial resolutions that optimize run time versus accuracy.

A prior study by Werginz *et al* analyzed the effect of multiple morphometric properties on extracellular stimulation thresholds using tracings from over 100 mouse *α*RGCs [47]. Soma diameter (15–25 *µ*m), dendritic field diameter (150–500 *µ*m), and axon hillock length (10–50 *µ*m) had no meaningful effect on activation thresholds. SOCB length (15–45 *µ*m) significantly influenced activation thresholds. In this study, we addressed three additional factors: dendritic arbor complexity, axon trajectory, and axon diameter.

RGC dendrites contain active ion channels that contribute to action potential generation [4, 48]. Prior models have made simplifications including eliminating dendrites or using an equivalent cylinder representation [21, 26]. Our sensitivity analysis showed that including a full branched dendritic morphology was important to produce reliable activation threshold maps. We limited our analysis to a single parasol cell in the mid-peripheral region [12, 29]. Prior work demonstrated that increasing dendritic field diameter from 150 *µ*m to 500 *µ*m had no significant influence on threshold [47]. Therefore, while including a full branched dendritic morphology is important for future models, the dendritic field diameter of parasol cells is unlikely to influence predictions. On the other hand, developing models of midget cells, which are prevalant in the foveal region and have much smaller dendritic arbors, should be investigated [49]. Unfortunately, no human midget cell tracings were available on the Neuromorpho database at the time of this publication.

RGC axons ascend from a soma in the inner retina to the nerve fiber layer, and the path they take varies naturally among cells [34]. Prior models have made various assumptions about the curvature of this path [9, 21, 35, 36]. Our sensitivity analysis revealed that RGC axon trajectory influences activation thresholds, specifically due to the orientation and distance of the SOCB in relation to the stimulating electrode. Regardless, the lowest thresholds always occurred when the electrode was above the SOCB, consistent with experimental results [10]. Furthermore, the average threshold profile is similar across all soma depths. These results demonstrate that future models should incorporate a range of axon trajectories or use the average trajectory and clearly state their assumptions.

The diameter of RGC axons (including their various subregions) has not been consistent in prior models. In our cable model, axon diameter had a minimal effect on activation threshold, but did influence action potential propagation. Prior models of intracellular current injection have similar findings. A model of neocortical pyramidal neurons established that axon hillock diameter can influence the efficacy with which a spike invades the soma [50]. Sheasby and Fohlmeister found that the diameter of the narrow region can influence whether an action potential will propagate uni-directionally or bi-directionally [5]. Importantly, cells with a high somatodendritic surface area and low axonal surface area can experience increased latency for an axonal spike to enter the soma or even failure of a spike to enter the soma altogether [5]. Our sensitivity analysis showed that for action potentials to propagate bi-directionally with extracellular stimulation, we must similarly avoid a large impedance (i.e. surface area) mismatch between the axon and soma. Based on experimental evidence, we believe that action potential propagation in both the orthodromic and antidromic direction is most realistic. Specifically, calcium imaging data shows somatic activation in response to axonally initiated spikes [51]. Therefore, the surface area of the axon (which is directly proportional to diameter) must be large enough to depolarize the somatodendritic surface area, given certain ion channel densities. For our particular cell tracing, an axon hillock diameter of at least 3 *µ*m and narrow region diameter of at least 0.8 *µ*m were necessary. Additionally, there was a small range of axon diameters that caused an axonal ‘echo spike’ (see figure 8(b)). It is difficult to garner experimental evidence about whether echo spikes occur in nature or are simply an artifact of the cable model. Therefore, it may be up to a modeler’s discretion whether to permit them in a simulation.

We also investigated two strategies for improving the computational efficiency of our RGC cable model. First, we optimized the temporal and spatial resolutions. The optimized section lengths are summarized in table 5. We found an ideal integration timestep of 5 *µ*s. Computation time is proportional to the product of Δ*t* and Δ*x*, allowing our optimized cable model to run 12.75 times faster without impacting threshold predictions. Given that the original full-resolution cable model took 112 min to run, this reduction can be significant depending on the number of neurons included in the simulation.

**Figure 10.**
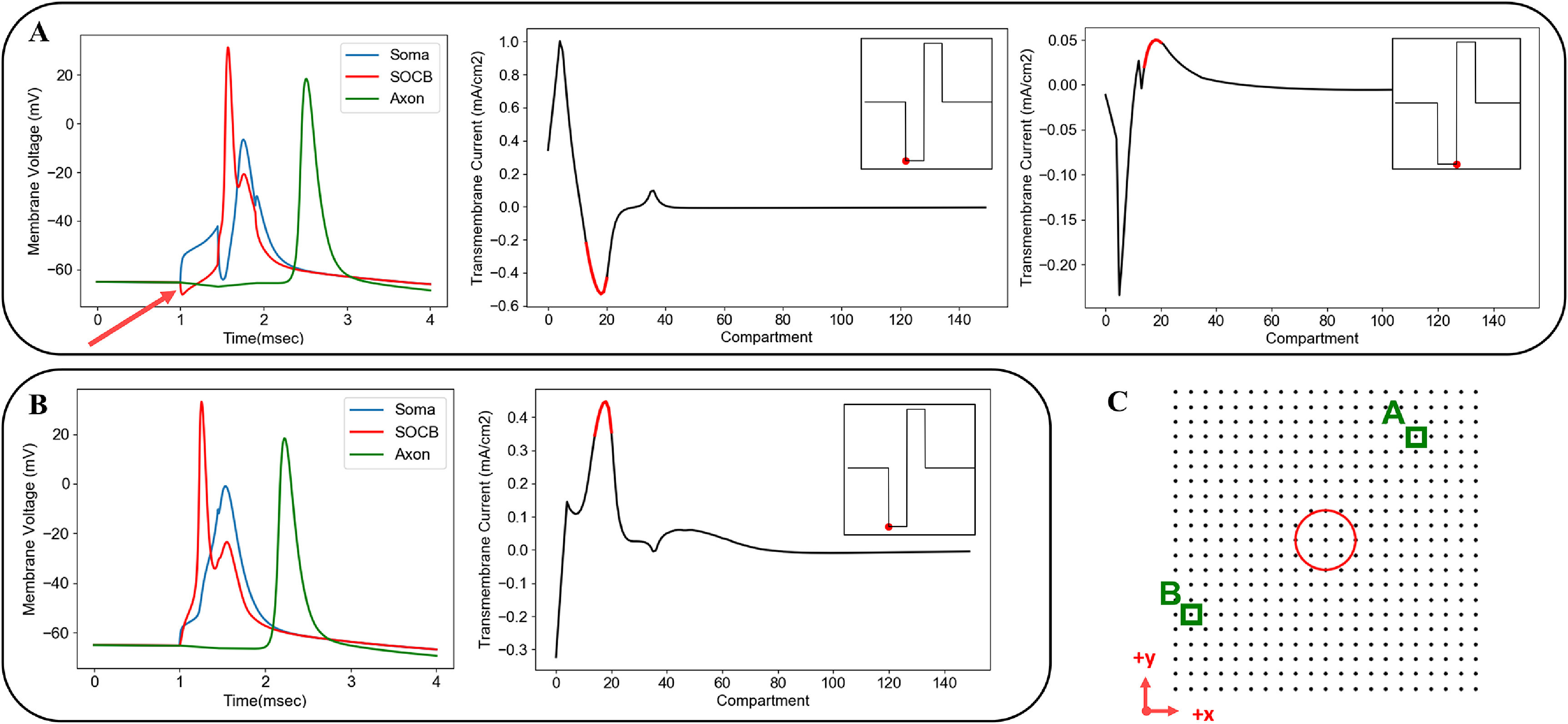
Example of RGC firing behavior that cannot easily be explained by the AF. (a) The left plot shows membrane voltage over time, with the red arrow indicating the initial hyperpolarization of the SOCB. Despite this hyperpolarization, the action potential still initiates in the SOCB. The middle plot shows the transmembrane current for each compartment at the time of the pulse onset (proportional to AF). The SOCB compartments are highlighted red, and SOCB current is negative. The right plot shows spatial transmembrane current at the end of the pulse. The SOCB compartments are highlighted red, and SOCB current has changed polarity. (b) Example where the SOCB is depolarized right away. The first plot shows membrane voltage over time. The second plot shows spatial transmembrane current at the pulse onset (proportional to AF), with the SOCB compartments highlighted in red. Immediately, SOCB current is positive. Overall, these plots highlight the challenges of using simplified threshold predictors derived from second spatial derivative of the extracellular potential field for non-uniform axons. (c) Location of the neurons in (a) and (b) with respect to the stimulating electrode.

Secondly, we found that simplified threshold prediction techniques could not reliably replicate the predictions generated with our full RGC cable model, in agreement with Werginz *et al* who observed that spikes are generally generated in the SOCB [36]. The varying ion channel densities and diameters between cell regions violate the assumptions of the AF framework that require homogeneous cell properties [41]. Figure 10 exemplifies how the AF, which is proportional to the instantaneous transmembrane current, does not clearly predict cell firing behavior. In figure 10(a), we see an example where the SOCB is hyperpolarized at the pulse onset, but the transmembrane current changes direction mid-pulse. By the end of the cathodic phase, current is peaking in the SOCB, instigating spike initiation. This behavior is caused by the high SOCB conductance (ion channel density) compared to neighboring regions. Figure 10(b) shows a comparison where the AF peak is located in the SOCB to begin with, and subsequent depolarization and spike initiation occur more rapidly. In general, the AF predicts the initial membrane response, and largely depends on where the cell is located in relation to the stimulating electrode (see figure 9(e)). However, for RGCs, this relationship could not reliably indicate what would happen by the end of the pulse, due to disproportionate axial current flow into the SOCB. We could not overcome these unpredictibilities, even by using a weighted AF as suggested by Esler *et al* [46]. Perhaps this disparity is because they drew conclusions based on a limited number of RGC locations, reducing the variability of AF shape.

There were several limitations of this work. First, we did not analyze the effects of changing regional maximum ion channel conductances. In all simulations, we used the constant values provided in table 3. These values are consistent with experimentally derived ion channel densities across four mammalian RGCs [6]. However, future work could include a more systematic analysis of ion channel conductance, spanning across the physiological range. Additionally, we limited our model to epiretinal stimulation, in which the stimulating electrode is located directly above the nerve fiber layer. In doing so, we considered only direct RGC activation, disregarding indirect activation via other cells (bipolar, amacrine) in the retinal network. For subretinal electrodes, it may be important to include a network model. Finally, RGC structural changes (e.g. dendritic field reduction, neurite sprouting) that may be induced by retinal degeneration were not included in our model [52, 53]. The presence and severity of these structural changes likely depends on the stage of disease progression and retinotopic location [52, 53].

For the purpose of this work, we calculated the extracellular potentials generated by the stimulating electrode using finite element analysis in COMSOL. In certain situations, a researcher may not have access to this software or may want to simplify their simulations. It is possible to calculate extracellular potential generated by a disc electrode using a simplified equation, as described by Wiley and Webster [54]. However, using this approach would disregard the effects of non-uniform conductivity for various tissue types. Alternatively, an open-source finite element analysis software (e.g. FEBio, Netgen) could be used.

## 5. Conclusion

Through this work, we aim to provide practical guidance for modeling the extracellular stimulation of RGCs to produce reliable and meaningful predictions. Additionally, we intend to increase the accessibility of these methods by sharing our code. Reliable computational models lay the groundwork for improving the performance of retinal prostheses. They allow for the design of novel stimulation paradigms and hardware, which could ultimately improve the quality of life for millions suffering from retinal degenerative diseases.

## Data Availability

All data that support the findings of this study are included within the article (and any supplementary files).
